# Development and Evaluation of Natural Hydrogel Nanocomposites for Skin Rejuvenation: Design and Optimization of Effective Formulations

**DOI:** 10.1111/jocd.70933

**Published:** 2026-05-29

**Authors:** Mahsa Ahankoob Nezhad, Akbar Esmaeili, Maryam Beladi

**Affiliations:** ^1^ Department of Chemical Engineering, NTC Islamic Azad University Tehran Iran; ^2^ Department of Microbiology, TeMS.C Islamic Azad University Tehran Iran

**Keywords:** antibacterial activity, biocompatible materials, chitosan coating, hydrogel nanocomposite, modified hyaluronic acid, skin rejuvenation

## Abstract

**Background:**

This study examines the development of innovative hydrogel nanocomposites from natural, biocompatible materials to rejuvenate skin and slow aging.

**Aim:**

To develop two distinct formulations to counteract internal and external aging factors.

**Methods:**

The first formulation comprises hyaluronic acid, starch, ascorbic acid, and selenium‐enriched microalgae, with chitosan and polyethylene glycol added. The second formulation combines chitosan, collagen, and *Origanum vulgare* extract, which are known for their restorative and anti‐aging properties. Research assessed polymer concentration, temperature, pH, and antibacterial properties, optimizing conditions using the RSM model.

**Results:**

High moisture absorption (85% and 75%) and strong antibacterial activity against gram‐positive bacteria (90% and 85%). Advanced statistical models confirmed the chemical structure and biocompatibility of the nanocomposites. The first formulation exhibited a particle size of about 148 nm, high moisture absorption, and significant antibacterial activity against *Staphylococcus aureus*.

**Conclusion:**

Both systems were validated through advanced analyses, demonstrating their efficacy for dermatological applications. The formulations were successfully incorporated into a semisolid cream with an oil‐in‐water emulsion base and exhibit promising physicochemical properties. This research highlights the potential of these nanocomposites for clinical applications in skin rejuvenation products. Future studies are encouraged to explore their use in other topical formulations, such as serums, lotions, and sunscreens.

**Abbreviations**
AA: Ascorbic acid
ANOVA: Analysis of variance

*B. subtilis*

:
*Bacillus subtilis*

BN:
*Bertholletia excelsa* extract (Brazilian Nut)
CCD: Central composite design
CCO: Chitosan/collagen/ *Origanum vulgare* extract
Ch: Chitosan
Co: Collagen
DLS: Dynamic light scattering

*ECM*

:
*Extracellular matrix*

*E. coli*

:
*Escherichia coli*

EDX: Energy dispersive X‐ray spectroscopy
E. faecalis: Enterococcus faecalis
FE‐SEM: Field emission scanning electron microscope
FESEM: Field emission scanning electron microscopy
FT‐IR: Fourier transform infrared spectroscopy
GAG: Glycosaminoglycan
GC–MS: Gas chromatography–mass spectrometry
HA: Hyaluronic acid
HBSAM‐C: Modified hyaluronic acid with BN/starch/ascorbic acid/chitosan coating

*L. acidophilus*

:
*Lactobacillus acidophilus*

*L. plantarum*

: *Lactiplantibacillus plantarum*
MBC: Minimum bactericidal concentration
O/W: Oil‐in‐water
OLS: Ordinary least squares
OVE:
*Origanum vulgare* extract

*P. aeruginosa*

:
*Pseudomonas aeruginosa*

PEG: Polyethylene gycol
PEGylated Chitosan: Chitosan coated and modified with polyethylene glycol
ROS: Reactive oxygen species
RSM: Response surface methodology

*S. aureus*

:
*Staphylococcus aureus*

SMLA: Selenium modified with *Lactobacillus acidophilus*

St: Starch
UV: Ultraviolet
UVR: Ultraviolet radiation
UV–Vis: UV–Visible spectroscopy
XRD: X‐ray diffraction
ZPA: Zeta potential analysis

## 1 Introduction

The skin, as the body's largest organ, plays a vital role in protecting against environmental factors and maintaining homeostasis. The skin aging process is a complex, multifaceted phenomenon influenced by both internal factors (such as genetic and hormonal changes) and external factors (including ultraviolet radiation, pollution, and oxidative stress). These factors lead to a decrease in collagen and elastin production, degradation of the extracellular matrix, increased inflammation, and ultimately result in wrinkles, sagging, and reduced skin elasticity [1, 2, 3, 4].

In this context, the use of active compounds with antioxidant, anti‐inflammatory, and collagen‐synthesis‐stimulating properties has gained attention as an effective strategy for skin rejuvenation. HA, a natural glycosaminoglycan, plays a crucial role in maintaining moisture retention and skin volume due to its high water‐absorbing capacity [5]. Modifying and restructuring HA to improve its physicochemical properties and enhance its biological activity has become an innovative approach in the production of skincare products [6].

Numerous studies have investigated the effects of hyaluronic acid and its derivatives on skin rejuvenation; however, research exploring the use of plant extracts to modify HA and enhance its properties remains limited [7, 8]. Specifically, the use of BN extract (B. excelsa), rich in antioxidants and nutrients, in combination with modified HA has been less studied [9].

In this study, two independent biofunctional delivery systems were designed to enhance skin regeneration and combat aging processes. The first formulation, referred to as HBSAM‐C (Modified Hyaluronic Acid with BN, Starch, Ascorbic Acid, and Chitosan Coating), is a hydrogel nanocomposite modified with polyethylene glycol. The second formulation, known as CCO (Chitosan, Collagen, *Origanum vulgare* extract), combines these components for their restorative and anti‐aging properties. The study investigates the effects of polymer concentration, temperature, pH, and antibacterial properties. Optimal conditions for both formulations were determined using the RSM model, yielding specific parameter values for HBSAM‐C and CCO. High moisture‐absorption capacity and strong antibacterial activity against Gram‐positive bacteria were observed. Advanced statistical models validated the chemical structure and biocompatibility of the nanocomposites, confirming their potential for practical dermatological applications.

**SCHEME 1:**
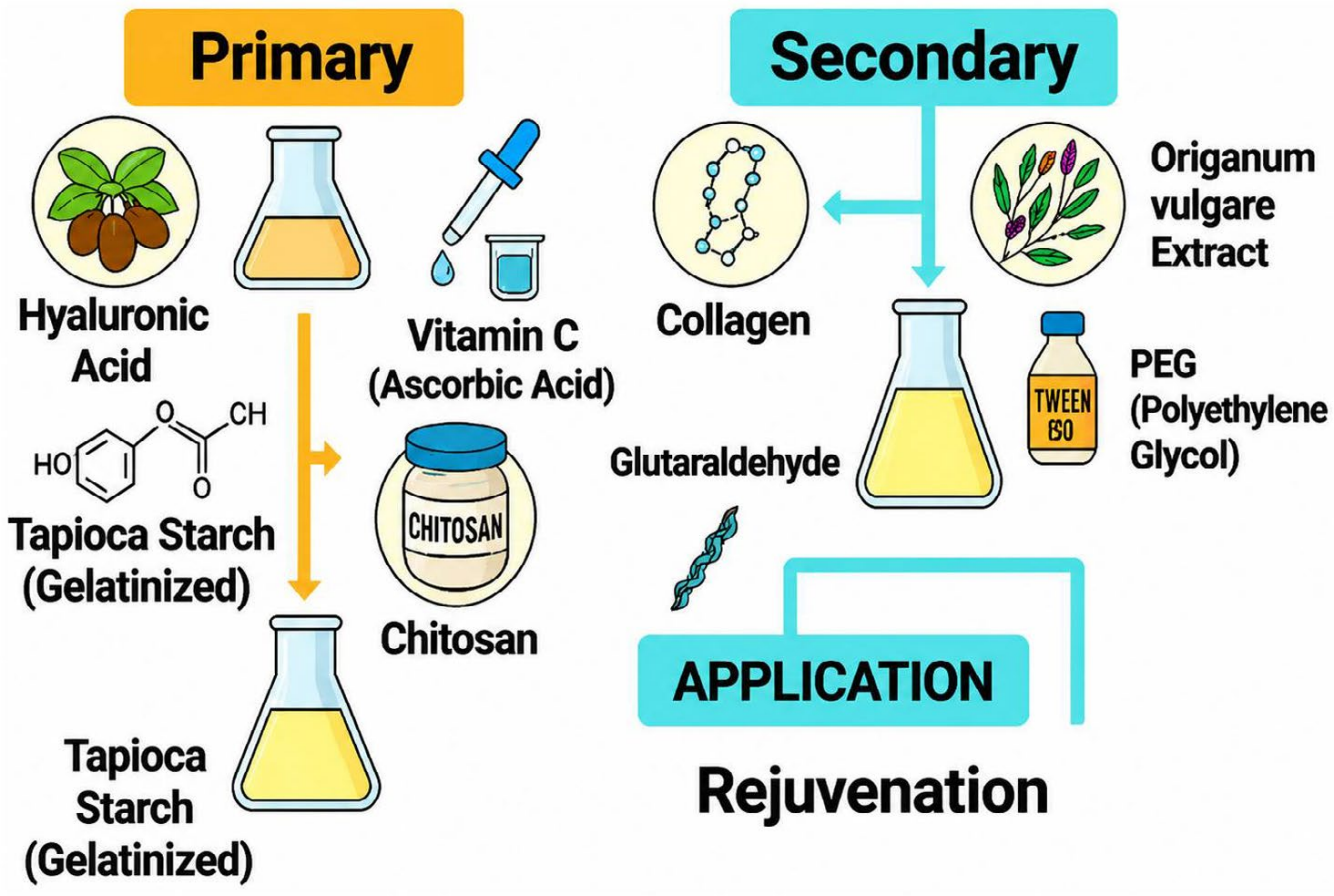
Synthesis of hydrogel nanocomposite.

Scheme 1 depicts the process of synthesizing a hydrogel nanocomposite that incorporates modified hyaluronic acid and extract derived from *L. acidophilus*. The final product is coated with chitosan, enhancing its stability and potential efficacy for skin rejuvenation applications. The design emphasizes the innovative combination of natural ingredients aimed at improving skin health and combating the effects of aging.

## 2 Materials and Methods

The first formulation involves preparing a hydrogel nanocomposite comprising HA, St, AA, SMLA, and Ch, which is PEGylated to enhance stability and control release. The second formulation combines Ch Co, and OVE to create a system with restorative and anti‐aging properties.

### 2.1 Materials

Hyaluronic acid (2%) and ascorbic acid (8%) were sourced from The Ordinary in Canada to ensure high quality and authenticity. Acetic acid (99.7% purity), Tween 80, glutaraldehyde (2%), polyethylene glycol (99% purity), starch (99% purity), sodium hydroxide (98% purity), and hydrochloric acid (37% purity) were obtained from Merck, Germany. These solvents and reagents are suitable for laboratory use. Chitosan with a purity of ≥ 90%, confirming its suitability for food‐related applications, was sourced from the *PINK WHITE ROSE* research center in Tehran, Iran.

### 2.2 Preparation of Oregano Extract ( *Origanum vulgare* )

Oregano leaves were collected in the spring of 2024 from the Shahou heights in Kurdistan Province, at approximately 2500 m above sea level, where the dominant vegetation included gorse and thyme. The collection was done manually using gardening scissors. After collection, the leaves were carefully washed and dried in the shade at room temperature. Identification of the oregano species was based on the plant's morphological characteristics, as described in the book “Flora Iranica.” For the extraction, 5 g of dried oregano leaf powder were soaked in 70% ethanol at a 1:2 weight‐to‐volume ratio for 48 h at room temperature, and then extracted using a water bath method. The resulting extract was filtered and concentrated via vacuum evaporation. The concentrated extract was stored at 2°C for use in preparing the nanocomposite [10].

### 2.3 Preparation of Brazilian Nut Extract ( *B. excelsa* )

Raw, organic BN was sourced from a reputable food importer (Salamat Kala Company, Tehran). The samples were vacuum‐sealed (from Brazil) and stored under standard conditions (at 4°C and relative humidity below 60%). To extract solvent‐soluble compounds from the BNs, 20 g of the nuts were weighed in a glass petri dish and ground using a laboratory grinder. The resulting powder was placed in a Soxhlet apparatus, and a mixture of 20 mL chloroform and 10 mL ethanol was used as the extraction solvent. The apparatus was covered with aluminum foil to prevent solvent evaporation. The extraction process was conducted for 20 min using the Soxhlet apparatus. After extraction, the solvent was evaporated under vacuum using a rotary evaporator, and the remaining extract was dried in an oven at 60°C for 24 h to remove any residual solvent. The resulting pure BN extract was wrapped in aluminum foil and stored in a freezer to prevent degradation from environmental factors [11].

### 2.4 Preparation of HBSAM‐C (Hyaluronic Acid, Starch, Ascorbic Acid, Selenium‐Enriched Microalgae, Chitosan‐Coated)

#### 2.4.1 Hydrolysis of Hyaluronic Acid (HA) With Sodium Hydroxide

HA, with a molecular weight of 766.6 g/mol and a density of 1.8 g/cm^3^, was dissolved in an ice bath (80%) using a 1 M sodium hydroxide solution at a ratio of 3.6 g or 4 mL. The hydrolysis reaction was conducted at pH 10 and 4°C for 2 h.

#### 2.4.2 Preparation of Modified Hyaluronic Acid (HA) With Brazilian Nut Extract

The selenium‐containing BN extract, extracted from *Lactobacillus* microorganisms using 70% ethanol via Soxhlet extraction, was added to the hydrolyzed hyaluronic acid. The weight ratio of hyaluronic acid to BN extract was set at 10:1 for the modification of hyaluronic acid [12].

#### 2.4.3 Preparation of Hyaluronic Acid (HA), B. excelsa, and Starch (St)

Tapioca starch (due to its suitable properties for the skin and soft texture) was gelatinized at a concentration of 3.8 g in a glass three‐necked flask at 90°C for 30 min. Gelatinization was done with continuous stirring (200 rpm) to prevent clumping. The ratios of starch to hyaluronic acid were set at 6, 4, 2, and 1, while keeping the hyaluronic acid amount constant.

#### 2.4.4 Preparation of Vitamin C/Starch/Hyaluronic Acid Modified With *B. excelsa*

For the preparation of the hydrogel nanocomposite, ascorbic acid (for greater stability in aqueous formulations) was used as a source of ascorbic acid at concentrations of 1%, 2%, 3%, and 4% by weight based on the total weight of polymers. Ascorbic acid was added to the polymer solution, and the mixture was ultrasonicated (40 kHz) for 15 min to ensure uniform distribution. The prepared suspension was then added to the gelatinized starch at 65°C and mixed for 30 min in a nitrogen atmosphere (to prevent oxidation). The polymerization reaction was initiated by adding 0.3 g of H_2_O_2_, and the hydrogel was dried in an oven at 60°C for 24 h.

### 2.5 Coating the Hydrogel Nanocomposite With Chitosan

#### 2.5.1 Coating Nanoparticles With Chitosan Polymer

A 100 mL solution of 2% acetic acid (2 g per 100 mL of distilled water, Merck) was prepared. Dissolving Chitosan Powder: 5.0 g of chitosan powder with 80% deacetylation (sourced from *PINK WHITE ROSE*) was added to the solution. This mixture was stirred at 60°C using a magnetic stirrer (IKA RW 20) at 300 rpm for 20 min until Fully Dissolved.

#### 2.5.2 Adding Hydrogel Nanocomposite

0.5 g of the hydrogel nanocomposite (synthesized in the first phase) was accurately weighed and added to the chitosan solution. The mixture was stirred at 65°C at 200 rpm for 20 min.

#### 2.5.3 Adding Glutaraldehyde

1.4 mL of a 2% glutaraldehyde solution was added to the mixture. The reaction was conducted at room temperature (approximately 25°C) with continuous stirring for 20 min.

#### 2.5.4 Separation and Drying of Coated Nanoparticles

The nanoparticles were separated from the medium using a magnetic field and washed twice with sterilized distilled water (1 mL per wash). After drying in an oven at 40°C for 24 h, the samples were analyzed using various instruments to evaluate the coating.

### 2.6 Pegylation of the Chitosan‐Coated Nanoparticles

#### 2.6.1 Preparation of Pegylation Solution

0.5 g of the coated nanoparticles was dissolved in 100 mL of sterilized distilled water. Subsequently, 0.5 g of polyethylene glycol (PEG 4000) was added to the mixture, which was stirred mechanically at 200 rpm for 10 min.

#### 2.6.2 Adding Tween 80

2 mg was added to the mixture. The mixture was heated to 65°C and stirred continuously for 1 h.

#### 2.6.3 Sonication

After the reaction period, the sample was sonicated at room temperature (25°C) in an anaerobic environment (under nitrogen) for 15 min to improve homogeneity.

#### 2.6.4 Separation and Drying of Nanoparticles

The samples were separated magnetically and washed twice with sterilized distilled water (1 mL each time). Finally, they were dried in an oven at 40°C, and glycerin (as a softener and moisturizer) was added to convert the product into a cream for final evaluation.

### 2.7 Preparation of CCO (Chitosan, Collagen, *O. vulgare* Extract)

#### 2.7.1 Preparation of Chitosan Solution

In the initial step, 0.125 g of chitosan with a deacetylation degree of 90% were dissolved in 25 mL of 1% (*v/v*) acetic acid solution with continuous stirring using a magnetic stirrer (200 rpm) at a stable room temperature (25°C) for 2 h. This process aimed to obtain a homogeneous, clear solution free of lumps, providing a suitable substrate for interpolymeric interactions.

#### 2.7.2 Separation of Chitosan and Collagen

Next, 0.125 g of native collagen (from Collageno, Iran) were gradually added to the chitosan solution. The mixing process continued under isotropic conditions with uniform stirring (200 rpm) at room temperature for 2 h to ensure complete dissolution, physical dispersion, and the formation of initial electrostatic interactions between the chitosan and collagen chains.

#### 2.7.3 Addition of *O. vulgare* Extract

To the resulting dual‐polymer solution, 2 mL of an ethanolic extract of *Origanum vulgare* was added. To optimize the dispersion of the active phenolic compounds from the extract within the biopolymer matrix, the mixture was treated in an ultrasonic bath for 20 min. This process enhanced the contact surface and facilitated the effective penetration of plant compounds into the polymer network.

#### 2.7.4 Centrifugation and Separation of the Active Phase

The resulting solution was centrifuged at 4000 rpm for 15 min at 4°C to separate suspended particles and stabilize the final composition. This step was designed to recover the solid phase and remove excess liquid effectively.

#### 2.7.5 Final Drying by Thermal Evaporation

The separated solid precipitate was placed in a 10 × 10 cm Pyrex glass container with a layer thickness of approximately 2 mm. Thermal drying was performed in an oven at 60°C for 24 h to remove any remaining moisture, stabilize the final composite structure, and prepare it for subsequent analyses [13, 14, 15].

### 2.8 Microbial Section

#### 2.8.1 Prepare the Materials and Equipment

The need 10 screw‐cap test tubes for separate or duplicate cultures. Additionally, three 500 mL Erlenmeyer flasks are required for larger volumes of culture medium, which can be used for liquid media. One 250 mL Erlenmeyer flask is necessary for solid culture media. You should also gather the required amounts of culture medium powders, including BHI Agar, MRS Agar, MHA Agar, and MRS Broth. Sterilized distilled water or laboratory‐grade deionized water is essential as well. Furthermore, a beaker, a precision scale for accurate powder measurement, a magnetic stirrer for mixing solutions, cotton and foil to cover the Erlenmeyer flasks containing culture media, a clamp to hold the Erlenmeyer flasks during heating, and a buret for precise measurement of distilled water are all needed.

#### 2.8.2 Preparation of Solid Culture Medium

For BHI Agar, dissolve 13 g of BHI Agar powder in 250 mL of sterile distilled water. For MRS Agar: Dissolve 17.05 g of MRS Agar powder in 250 mL of sterilized distilled water. For MHA Agar: Dissolve 1.5 g of agar and 8.5 g of MHA powder in 250 mL of sterilized distilled water. Each solid culture medium should be stirred thoroughly and gently with a magnetic stirrer until the powder is fully dissolved. Cover the Erlenmeyer flasks with cotton and foil, and place them in a water bath (slowly and carefully) until the culture medium is completely melted. After complete melting, remove the culture medium from the water bath and allow it to cool.

#### 2.8.3 Preparation of Liquid Culture Medium

For MRS Broth, dissolve 10.44 g of MRS Broth powder in 200 mL of sterile distilled water, stirring thoroughly until completely dissolved. Finally, cover the Erlenmeyer flask with cotton and foil.

#### 2.8.4 Bacterial Culture Methods

In this study, two strains of Gram‐positive bacteria, *L. acidophilus* and *L. plantarum* , were cultured. Culturing Lactobacillus acidophilus.

##### 2.8.4.1 Preparation of Inoculum

Sterilize a swab by exposing it to a flame. Then, inoculate the swab with bacteria.

##### 2.8.4.2 Culturing

The inoculated swab was then cultured on solid media, MRS agar and BHI agar, using a four‐step culturing method. Anaerobic Conditions: The cultured plates were placed in an anaerobic jar. A lit candle was then placed inside, and the jar was immediately sealed to create anaerobic conditions. Incubation: The jars were incubated for 48 h at 38°C in an incubator.

#### 2.8.5 Culturing L. plantarum

##### 2.8.5.1 Preparation of Inoculum

Sterilize a swab by exposing it to a flame. Then, inoculate the swab with bacteria.

##### 2.8.5.2 Culturing

The inoculated swab was then cultured on BHI agar using the lawn culture method.

##### 2.8.5.3 Anaerobic Conditions

The cultured plates were placed in an anaerobic jar. A lit candle was then placed inside, and the jar was immediately sealed to create anaerobic conditions.

##### 2.8.5.4 Incubation

The jars were incubated for 48 h at 38°C in an incubator. After the incubation period, the plates were examined for colony growth. These methods were used to optimize bacterial growth and obtain visible colonies suitable for further studies.

#### 2.8.6 Gram Staining Preparation of Slide

In Step 5 of Gram staining, prepare a clean slide and add a drop of distilled water. Use a swab to take a colony of bacteria and spread it in the water to create a smear. Allow the slide to dry at room temperature, then fix it by heating it three times to kill the cells. Stain the slide with crystal violet for 60 s, then rinse with distilled water. Apply Lugol's solution for 5 s, wait 45 s, and rinse again. Use a decolorizer to make Gram‐negative bacteria colorless while Gram‐positive bacteria remain purple. Finally, stain with safranin for 1 min, rinse, and observe under a microscope; purple indicates Gram‐positive bacteria, while red or pink indicates Gram‐negative bacteria [3, 11, 16, 17].

#### 2.8.7 Bacterial Growth Curve Based on Optical Density ( **OD** )

The growth curve of *L. plantarum* was examined in MRS broth. Initially, a suspension of *L. acidophilus at* half McFarland (approximately 10^8^–10^9^ bacterial cells) was cultured in MRS Broth until the medium was fully turbid. This step was designed to create optimal conditions for *L. plantarum growth*. Then, a specific volume of the prepared *L. plantarum* suspension was inoculated into the medium containing *L. acidophilus* . The incubator temperature was set at 37°C during this experiment. The optical density (OD) of the samples was measured at 530–540 nm using a UV–Vis spectrophotometer at various time points. Measurements were repeated at the first moment (inoculation time) and then every 2 h for 72 h. During this period, the samples were kept in the incubator and removed at specified time points for optical density measurements. Finally, the collected optical density data were plotted in Excel to create the growth curve for *L. plantarum* .

#### 2.8.8 Adding Brazilian Nut Extract to Culture Medium

Initially, *L. acidophilus* was cultured in MRS broth until a half McFarland concentration was reached. The optical density (OD) of the sample was measured at time 0 using a spectrophotometer (model UV‐1800, Shimadzu) at 600 nm. Then, 1 μL of the desired extract was carefully added to the culture medium using a sampler, and the sample was placed on a shaker to ensure uniformity. Immediately after that, 1 μL of the new culture medium was taken, and its OD was measured simultaneously. This process was repeated to examine the effects of the extract on bacterial growth throughout the experimental period. The samples were placed in an anaerobic incubator at 30°C for at least 48 h to allow for complete bacterial growth. After this period, the samples were removed from the incubator and centrifuged at 8000 RPM for 10 min to separate the microbial suspension into two distinct phases: a supernatant (the upper phase, containing the extract and soluble materials) and a sediment (the lower phase, containing bacterial cells). This separation was crucial for a more detailed examination of the extract's effects on bacterial cells [18].

### 2.9 Antibacterial Activity

#### 2.9.1 Evaluation of Antimicrobial Effects of Nanoparticles Using the Disk Diffusion Technique

##### 2.9.1.1 Agar Diffusion Method

The antimicrobial activities of the primary and secondary nanoparticle composites were determined using the disk diffusion method (Disc diffusion assay, NCCLS, 1997). The nanoparticles were dissolved in water at a final concentration of 250 μg/mL as the stock solution and filtered through a 0.45 μm Millipore filter for sterilization. Ultrasonication was used to ensure complete dissolution. Then, 250 μL of the bacterial suspension with a half McFarland dilution (CFU 1.5 × 10^8^/mL) of the bacteria (*Staphylococcus aureus, Enterococcus faecalis, Escherichia coli*, and *Pseudomonas aeruginosa* ) was inoculated onto Mueller‐Hinton agar (MHA) in three directions: vertical, horizontal, and diagonal, ensuring that the entire plate surface was covered with a uniform microbial layer. Disks containing nanoparticles (6 mm in diameter) were placed on the plate, spaced 2.5 cm apart from each other and the edges of the plate. Positive controls included disks containing antibiotics such as gentamicin and ciprofloxacin for *Staphylococcus aureus* and *Enterococcus faecalis* , as well as gentamicin, ciprofloxacin, and cefepime for *Pseudomonas aeruginosa* and *Escherichia coli* . After incubation for 24 h at 37°C, the inhibition zones were measured, and the results were evaluated according to CLSI guidelines to assess sensitivity. The diameter of the inhibition zone was used as a measure of antimicrobial activity, and all tests were performed in triplicate [18].

#### 2.9.2 Determining the Minimum Inhibitory Concentration (MIC)

In this method, the minimum inhibitory concentration (MIC) for microorganisms sensitive to the nanoparticles was calculated using the Micro‐Well dilution method. In this study, serial dilutions were performed in several test tubes to determine the MIC. For this purpose, 10 test tubes containing Mueller‐Hinton agar (MHA) at 250 μg/mL were prepared. The first tube contained 250 μL of the stock concentration, and serial dilutions were made to 8 concentrations (250, 125, 62.5, 31.2, 15.6, 7.2, 3.9, and 1.9 μL/mL) in subsequent tubes. A 10 μL bacterial suspension with a 0.5 McFarland dilution was added to each tube except for tube number 9. Thus, a serial dilution was prepared through tube 9, with each tube containing half the concentration of the previous one. Tube 9 served as a negative control containing culture medium and nanoparticles, while tube 10 served as a positive control containing culture medium and bacteria. Then, the tubes were incubated at 37°C for 24 h. Microbial growth was indicated by turbidity in the tubes. To confirm the MIC, the MBC method was performed by culturing the tubes of interest on Nutrient Agar (NA). The minimum concentration of nanoparticles required to inhibit the growth of each microorganism is defined as the MIC. The last dilution that kills 99% of the bacteria is considered the MBC. All tests were repeated three times [2].

## 3 Result and Discussion

### 3.1 Optimization

This section outlines the statistical analysis methods used in our research, ensuring reliable and meaningful comparisons of data. We employed Response Surface Methodology (RSM) based on Central Composite Design (CCD) to analyze the simultaneous effects of various factors influencing biogenic nanoparticle synthesis. A quadratic polynomial regression model was used, and ANOVA confirmed its validity.

### 3.2 Two Samples Were Designed for Analysis

The HBSAM‐C sample, consisting of 19 experiments with variables like hyaluronic acid concentration and temperature, and the CCO sample, also with 19 experiments focusing on the chitosan‐to‐collagen ratio and oregano extract concentration.

Analyses were conducted using Python 3.11, evaluating model fit indices such as the coefficient of determination (*R* ^2^), statistical significance (*p*‐value), and residual analysis for normality and outliers. This statistical framework supports accurate predictions for optimal formulation design [19, 20, 21].

#### 3.2.1 Results of ANOVA

The comparative evaluation of two nanocomposites using Response Surface Methodology (RSM) revealed significant differences in factor effects. For the HBSAM‐C sample, three independent variables—HA concentration, extract percentage, and temperature—significantly influenced particle size (*p* < 0.0001). The model showed a strong fit with no significant Lack of Fit (*p* = 0.1482), highlighting HA's dominant role in controlling particle morphology.

In contrast, the CCO sample, while having a suitable model fit, demonstrated different influencing factors. The concentration of *Origanum vulgare* extract (*p* = 0.0034) and the CS:Col ratio (*p* = 0.0345) were key determinants for size, while temperature had no significant impact (Table 1).

**TABLE 1:** Statistical analysis of variance (ANOVA) for HBSAM‐C and CCO nanocomposites.

Source		*Df*		Sum of squares		Mean square		*f*		*p*	
HBSAM‐C	CCO	HBSAM‐C	CCO	HBSAM‐C	CCO	HBSAM‐C	CCO	HBSAM‐C	CCO	HBSAM‐C	CCO
Model	Model	5	5	2354.76	157.60	470.95	31.52	29.38	4.11	< 0.0001	0.0271
A (HA content)	A (CS:Col ratio)	1	1	684.21	45.91	684.21	45.91	42.69	5.98	< 0.0001	0.0345
B (Extract)	B (Extract)	1	1	497.32	111.70	497.32	111.70	31.01	14.56	< 0.0001	0.7969
C (Temperature)	C (Temperature)	1	1	378.67	0.54	378.67	0.54	23.63	0.07	0.0002	0.6053
AB	AB	1	1	108.57	2.18	108.57	2.18	6.77	0.28	0.0187	0.6394
A^2^	A^2^	1	1	686.00	1.79	686.00	1.79	42.80	0.23	< 0.0001	—
Residual	Residual	10	10	160.29	76.70	16.03	7.67	—	—	—	0.2668
Lack of Fit	Lack of Fit	5	5	112.45	51.13	22.49	10.23	2.38	1.59	0.1482	—
Pure Error	Pure Error	5	5	47.84	25.57	9.57	5.11	—	—	—	—
**Total**	**Total**	15	15	**2515.05**	**234.30**	—	—	—	—	—	—

**FIGURE 1:**
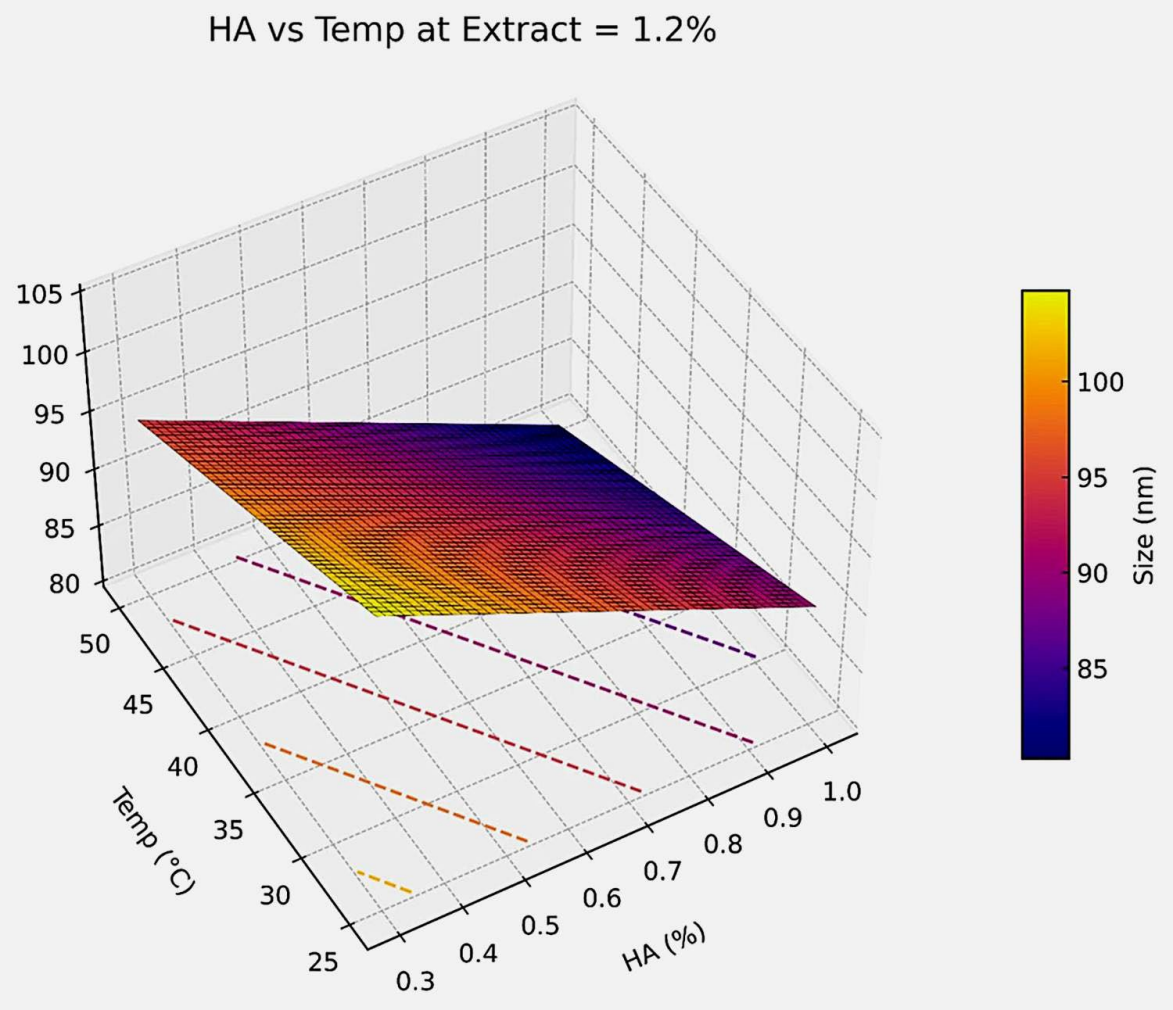
Response surface plot of nanoparticle Size as a function of HA concentration and reaction temperature.

**FIGURE 2:**
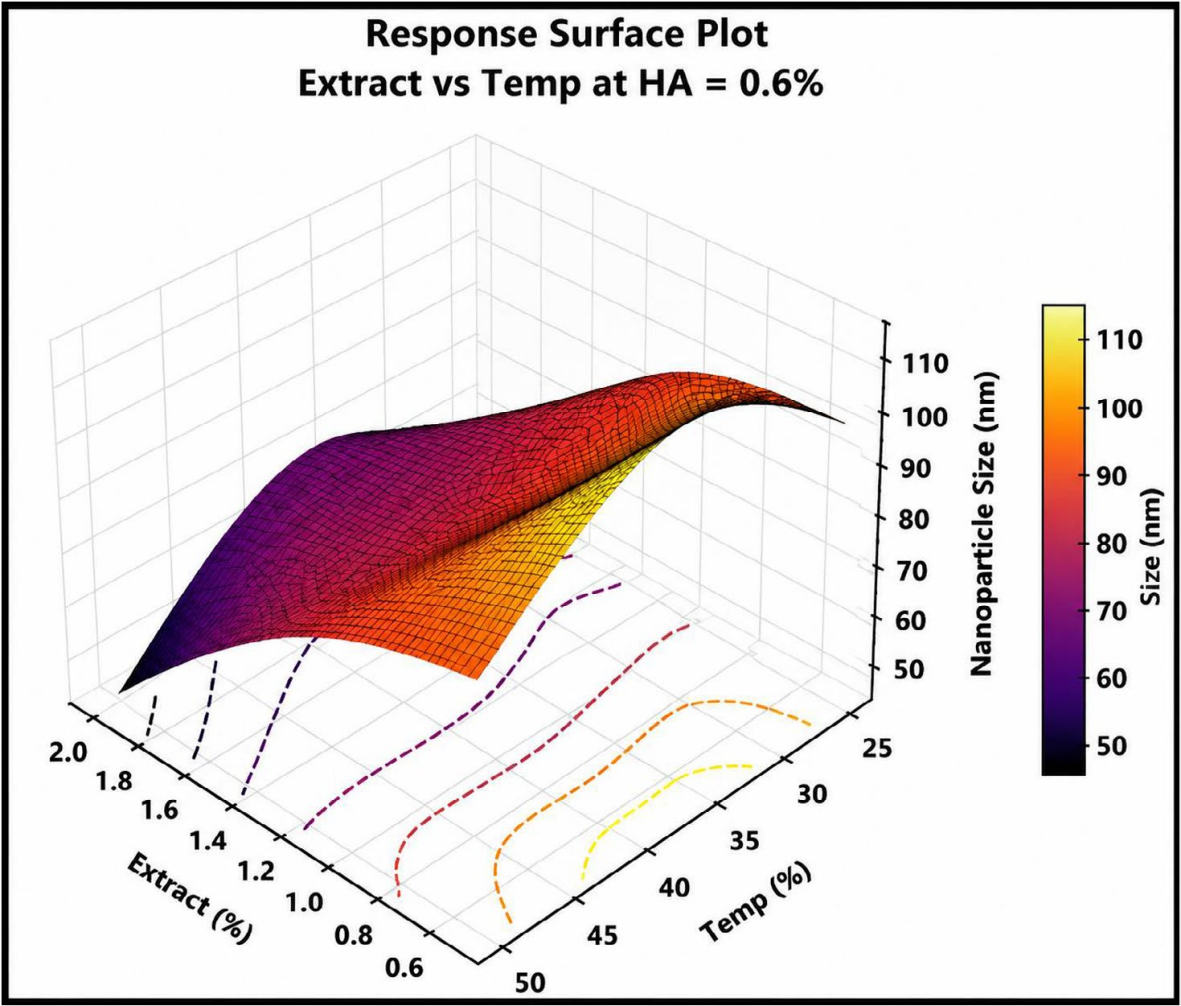
Response surface analysis of nanoparticle size as a function of oregano extract concentration and reaction temperature.

**FIGURE 3:**
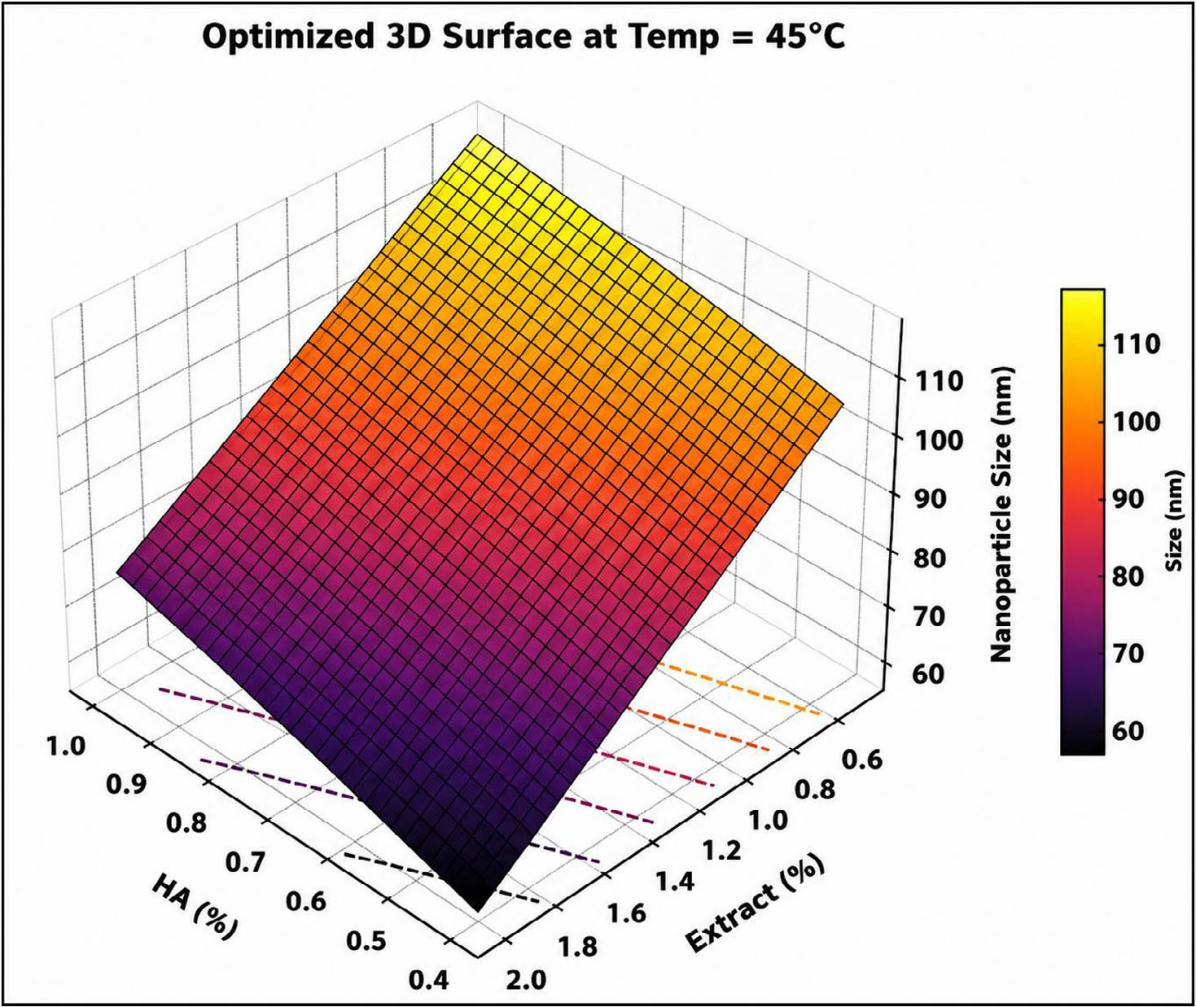
3D Response surface plot analyzing the Simultaneous effects of HA concentration and oregano extract on nanoparticle Size at a constant temperature of 45°C.

This study compares two nanocomposites, HBSAM‐C and CCO, using Response Surface Methodology (RSM). Figure 1 shows that increasing HA concentration raises nanoparticle size, especially at lower temperatures, while higher temperatures decrease size, indicating a negative interaction. Figure 2 shows that initial increases in oregano extract concentration reduce particle size, but further increases lead to aggregation, with optimal particle size achieved around 35°C. Figure 3 confirms that higher HA concentrations lead to larger particles, whereas higher extract concentrations stabilize and reduce particle size.

Overall, these findings emphasize the critical role of formulation variables in nanoparticle characteristics, with HBSAM‐C showing structural control and CCO focusing on biological optimization for skin applications.

ANOVA results (Table 1) confirmed strong model fits for both systems, with HBSAM‐C showing a highly significant F‐value for HA and its interactions, whereas CCO showed notable effects of CS:Col ratios but less from other factors. The RSM models, fitted as second‐degree polynomial regressions, demonstrated high predictive accuracy (*R* ^2^ = 0.997 for HBSAM‐C and *R* ^2^ = 0.982 for CCO).

The final equations for nanoparticle size in both models revealed that HA concentration positively influenced size, while BN extract negatively affected it. Temperature had a dual effect: initially reducing size, then increasing it beyond a threshold [5].

The general form of this model is expressed as (Equation (1)).
(1)

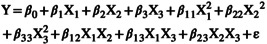

The final equation of the model, derived from the actual coefficients obtained from fitting the experimental data of the first sample, is presented as (Equation (2)).

(2) $$\begin{array}{c} \text{Size}\left(\mathrm{nm}\right)=32.31+42.15\cdot \mathrm{HA}-38.13\cdot \text{Extract}+4.51\cdot \text{Temp}\\ -6.14\cdot {\mathrm{HA}}^{2}+0.86\cdot {\text{Extract}}^{2}-0.06\cdot {\text{Temp}}^{2}+7.26\cdot \mathrm{HA}\cdot \text{Extract}\\ -0.33\cdot \mathrm{HA}\cdot \text{Temp}+0.005\cdot \text{Extract}\cdot \text{Temp} \end{array}$$

Equation (2) includes linear, nonlinear (quadratic), and interaction effects of the three variables, each playing a distinct role in the final nanoparticle size. The analysis of the model coefficients revealed that HA has a strong positive impact on particle size; as its concentration increases, nanoparticle size significantly enlarges, which can be attributed to increased polymer network density and inter‐chain aggregation [22]. Conversely, the BN extract exhibits a significant negative coefficient, indicating a reducing role on particle size, likely due to the surface activity and stabilizing properties of its phenolic compounds [4]. The effect of temperature appeared dual; its linear component is positive, while the quadratic component is harmful, suggesting that increasing temperature initially decreases particle size, but beyond a certain threshold, particle growth resumes due to increased molecular motion [18]. Additionally, the interaction between HA and the extract (coefficient + 7.26) indicates that the simultaneous presence of high concentrations of both can weaken the extract's reducing effect, suggesting a positive but non‐enhancing interaction [1]. The interaction between HA and temperature is inverse, suggesting that simultaneous increases in both factors may inhibit particle growth. On the other hand, the interaction between temperature and extract did not show a significant statistical impact and can be omitted from simplified models.

(3) $$\begin{array}{c} \text{Size}\left(\mathrm{nm}\right)=144.77+4.84A-35.12B-1.04C+18.75A2\\ +3.88B2+0.00\text{2C}2+1.67\mathrm{AB}-0.11\mathrm{AC}+0.05\mathrm{BC} \end{array}$$

In contrast, the (Equation (3)) of the Model, derived from the actual coefficients obtained from fitting the experimental data of the second sample.

HBSAM‐C Model: Optimal conditions for small nanoparticles involved low HA concentrations, high extract percentages, and a temperature of 37°C, resulting in an average size of ~90 nm. CCO Model: Larger particle sizes were linked to higher CS: Col ratios, while increased extract concentrations reduced size.

Overall, the RSM analysis emphasized the importance of formulation variables in controlling nanoparticle characteristics, with the HBSAM‐C model providing a more physicochemically controlled approach and the CCO model offering biological optimization for skin applications.

### 3.3 Morphology and Size of Nanoparticles

**FIGURE 4:**
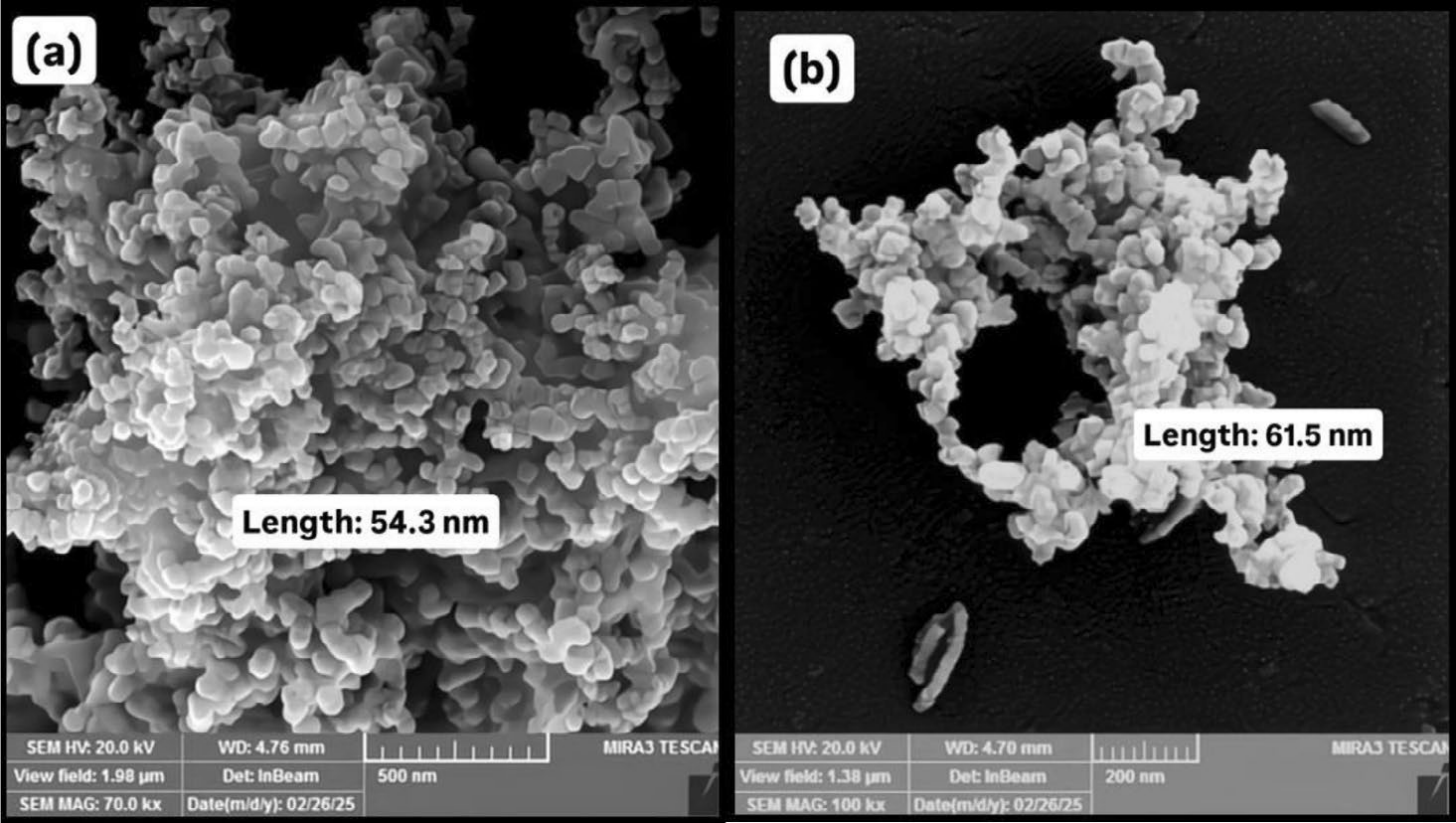
FE‐SEM images of chitosan nanoparticles and plant extract‐loaded nanocomposites at magnifications of 70 000× and 100 000×.

**FIGURE 5:**
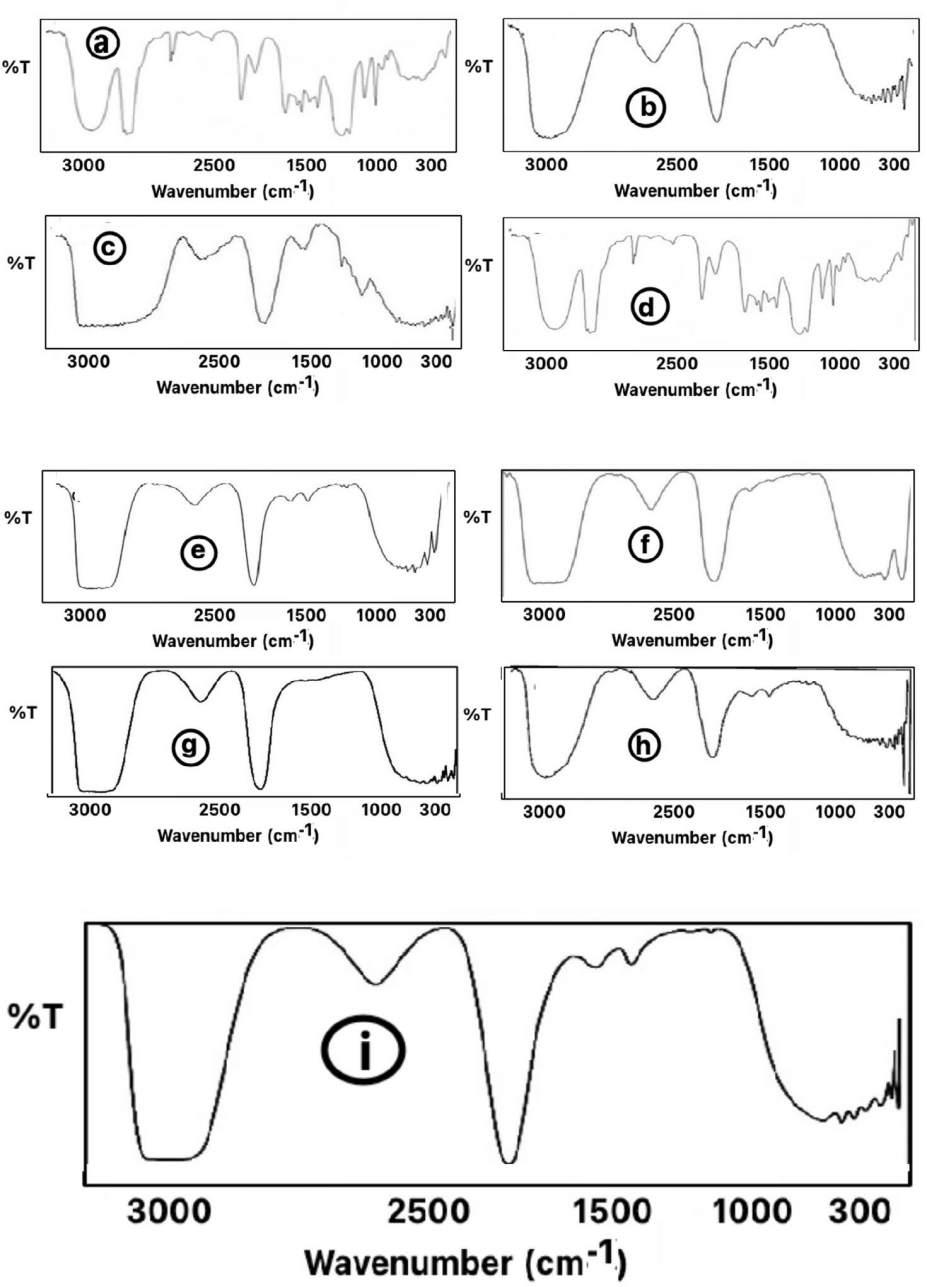
Compositions of various extracts and materials. a: BN (brazilian nut extract); b: Ch (chitosan solution); c: HA (hyaluronic acid) modified with BN derived from Mmicroorganisms; d: Modified HA with BN/AA (ascorbic acid); e: Modified HA with BN/AA/St (starch); f: Modified HA with BN/AA/St coated with Ch; g: Chitosan Pegylation with PEG (polyethylene glycol) and the Final HBSAM‐C nanocomposite; h: Ch/Co (chitosan/collagen solution); i: Ch/Co/OVE (chitosan/collagen/ *O. vulgare* extract) and the final CCO nanocomposite.

**FIGURE 6:**
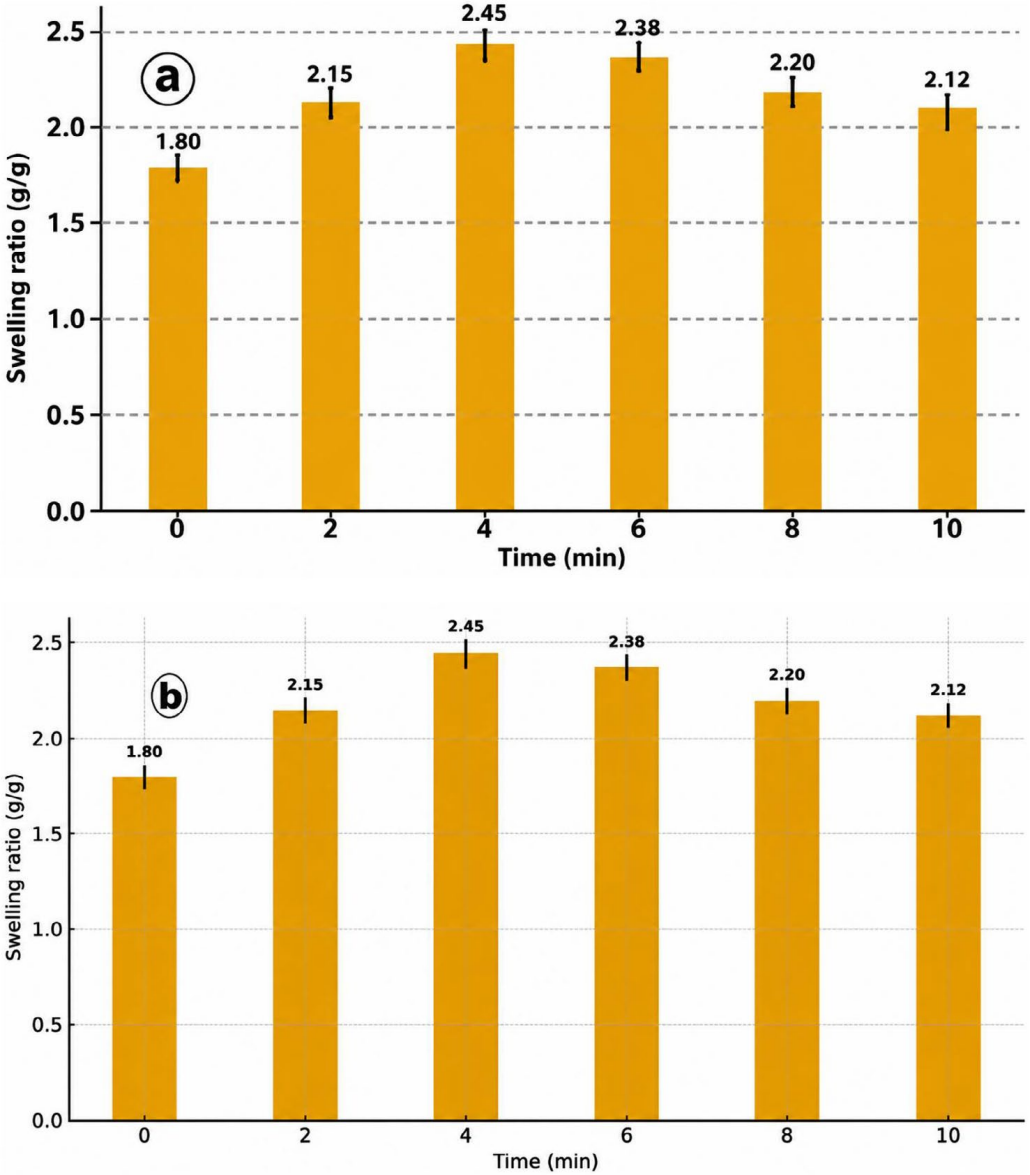
(a) Related to the HBSAM‐C nanocomposite. (b) Related to the CCO nanocomposite.

**FIGURE 7:**
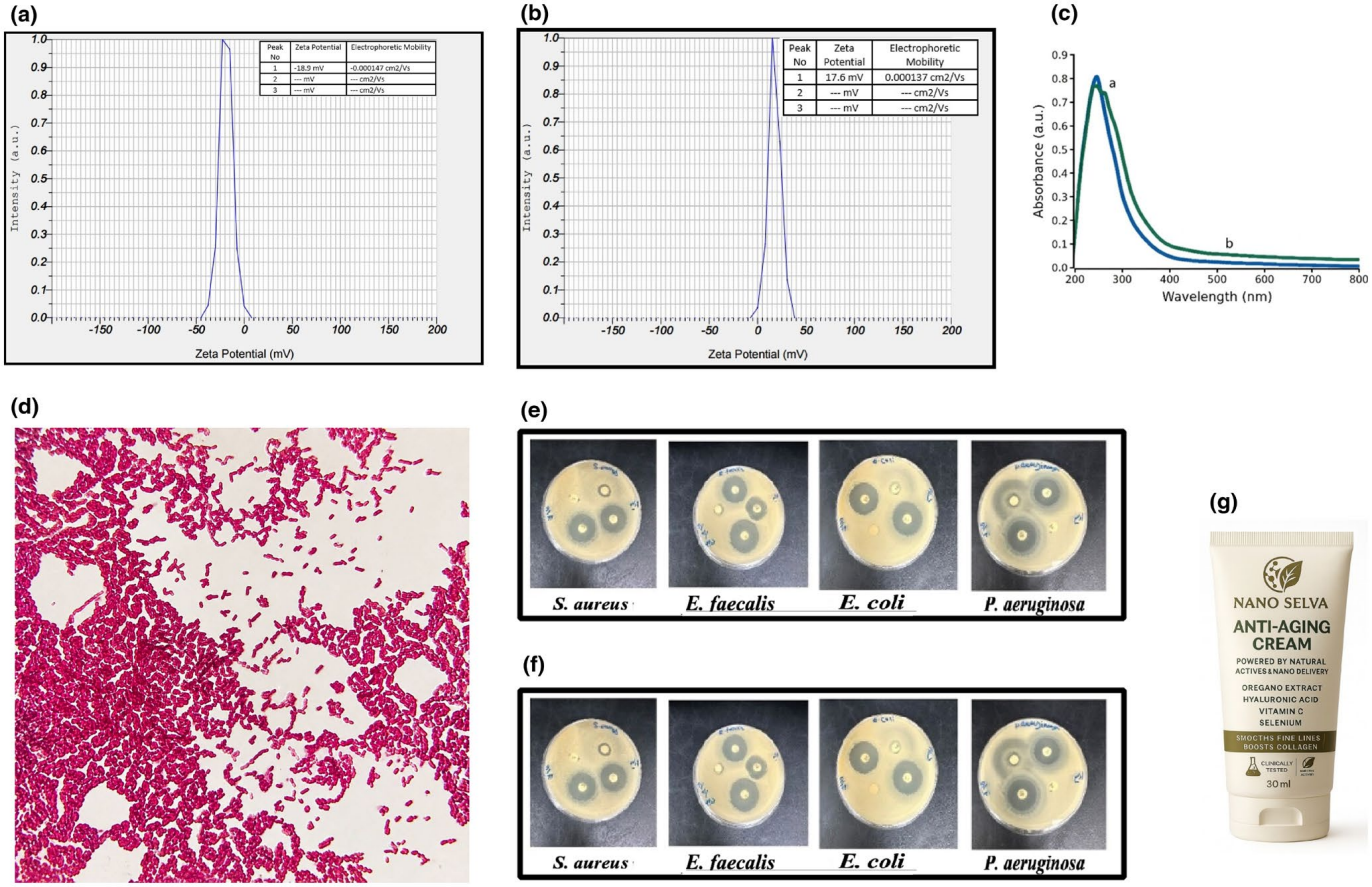
(a) Zeta potential of HBSAM‐C nanocomposite. (b) Zeta potential of CCO nanocomposite. (c) Absorbance spectrum of ascorbic acid and oregano extract in HBSAM‐C and CCO nanocomposites. (d) Gram staining of probiotic bacteria. (e) Antibacterial activity of synthesized nanocomposites against selected bacterial Strains. Antibiogram of HBSAM‐C nanocomposite. (f) Antibacterial activity of synthesized nanocomposites against selected bacterial strains. Antibiogram of CCO nanocomposite. (g) Nanocomposite ointment based on nanohydrogel.

The morphology and size of the nanoparticles were examined using a FE‐SEM MIRA3 from TESCAN, Japan. SEM images at magnifications of 70 000× and 100 000× (Figure 4a) provided clear insights into the structures of pure chitosan nanoparticles and chitosan‐based (Figures 5, 6, 7)nanocomposites loaded with BN extract derived from *Lactobacillus*.

The results in Figure 4a indicated that the chitosan nanoparticles exhibited a spherical, relatively uniform structure, with an average particle size of 47–58 nm. In contrast, the plant extract‐loaded nanoparticles (Figure 4b) displayed a rougher, more irregular structure due to the inclusion of phenolic and antioxidant compounds in the extract, resulting in an average diameter of 50–65 nm. This change in size and morphology reflects effective adsorption of the extract onto the nanoparticle surfaces and alterations in their surface energy.

The spherical morphology of the nanoparticles is crucial for enhancing their ability to penetrate skin tissues. Spherical nanoparticles increase the contact surface area, improve the release of active compounds, and facilitate deeper absorption into skin layers, potentially contributing to increased collagen synthesis and improved skin elasticity.

The study found that chitosan nanoparticles synthesized via self‐emulsification ranged in size from 10 to 1000 nm, depending on processing conditions. This aligns with findings by Ghadi et al., which reported magnetic nanoparticles coated with chitosan ranging from 10 to 80 nm. The current study's synthesized particles fall within an optimal size range, making them suitable for biomedical applications, particularly in skin rejuvenation.

Overall, the combination of a spherical structure, appropriate size, and the active surface of the nanoparticles, along with the loading of bioactive compounds such as BN tract, positions the designed nanocomposite as a promising option for targeted release and therapeutic applications in skin treatments [23, 24].

### 3.4 Fourier Transform Infrared Spectroscopy (FT‐IR)

Fourier Transform Infrared Spectroscopy (FTIR) is essential for identifying functional groups and analyzing interactions in multi‐component systems, particularly in therapeutic and cosmetic formulations like anti‐aging creams. This study evaluated FTIR spectra from five skin rejuvenation formulations to confirm compound integrity and assess interactions with the base matrix. Figure 5a shows C–H stretching bands in the range of **2850–2920 cm** ^**−1**^ and a distinct ester carbonyl band around **1740 cm** ^**−1**^. These peaks confirm the presence of saturated and non‐polar fatty compounds, which enhance skin absorption and provide surface softness. Figure 5b reveals broad bands between **3200 and 3400 cm** ^**−1**^, corresponding to hydroxyl and primary amine groups in chitosan. The peaks at **1650** and **1570 cm** ^**−1**^ represent amide bands, indicating chitosan's semi‐crystalline structure, which offers biocompatibility and protective benefits for the skin. Figure 5c displays a broad peak around **3322 cm** ^**−1**^, attributed to overlapping O–H and N–H vibrations. This suggests a network rich in hydrogen bonds within the nanocomposite matrix. The peak at **1639 cm** ^**−1**^ corresponds to C=O (Amide I) stretching and indicates stabilization of the amide structure, contributing to the overall stability of the formulation. Figure 5d shows a broad peak at **3480 cm** ^**−1**^, indicative of O–H vibrations and hydrogen bonding between hyaluronic acid and the BN extract. The peak at **1638 cm** ^**−1**^ corresponds to amide groups, while vibrations around **1100 cm** ^**−1**^ indicate a modified HA network, enhancing the controlled release and stability of ascorbic acid. Low‐frequency peaks (557–476 cm^−1^) may reflect structural changes due to interactions with antioxidant compounds. Figure 5e shows a strong peak at **3473 cm** ^**−1**^, indicating O–H stretching and hydrogen bonding interactions in the HA‐starch‐polyphenol network. The peak at **1638 cm** ^**−1**^ relates to C=O (Amide I) vibrations, while peaks at **1392** and **1276 cm** ^**−1**^ reflect C–O and C–O–C vibrations, reinforcing the presence of glycosidic bonds in starch and indicating structural continuity and stability of the nanocomposite matrix. The FTIR spectrum of the HBSAM‐C nanocomposite (Figure 5f) shows a broad network of O–H and N–H in the range of 3320–3490 cm^−1^ and Amide I peaks at 1633–1639 cm^−1^, confirming the presence of hydrogen bonds and covalent interactions with phenolic/ascorbic compounds. The C ≡ C/C ≡ N peaks (2080–2078 cm^−1^) indicate bioactive modification and enhanced network stability. In contrast, the CCO nanocomposite (Figure 5g) features broader –OH/–NH bands (3200–3580 cm^−1^) and Amide I/II peaks (1636–1570 cm^−1^), indicating protein‐polysaccharide interactions that provide greater flexibility. Figure 5h shows an increase in the Amide I and II bands, indicating improved structural integrity and elasticity.

Additionally, Figure 5i shows strong hydrogen bonding between chitosan, collagen, and phenolic compounds, confirming the stability of these interactions. Overall, the HBSAM‐C nanocomposite exhibits superior chemical stability and antioxidant properties, while the CCO nanocomposite demonstrates enhanced mechanical strength and biocompatibility for tissue regeneration. Overall, FTIR analysis provides valuable insights into the functional efficacy and interactions of these formulations, confirming their potential for skin rejuvenation [25].

### 3.5 Swelling Ratio of Hydrogel Nanocomposites

The swelling ratio is a key parameter in designing biocompatible hydrogel nanocomposites, as it indicates the polymer network's capacity to absorb water and biological fluids. This property is crucial for applications such as skin rejuvenation, as it directly affects the release rate of active compounds and the compatibility with body tissues. The synthesized nanocomposites are designed to enhance biocompatibility while providing a porous structure for high moisture absorption.

Figure 6a,b shows the swelling behavior, which increased rapidly from 1.80 to 2.45 g/g in the first 4 min, stabilizing at 2.12 g/g by the 10th minute. This two‐phase pattern results from the high accessibility of hydrophilic groups in starch and hyaluronic acid, leading to immediate water absorption and the formation of hydrogen and electrostatic bonds between chitosan, HA, and antioxidants. PEG plays a crucial role by preventing sudden network collapse and controlling swelling.

The two‐stage pattern is due to synergistic interactions among components: PEG and chitosan prevent network failure, while vitamin C and phenolic compounds enhance water absorption. This behavior creates an effective combination of immediate hydration and sustained moisture for skin rejuvenation formulations [26].

**Sample 9b described** the swelling ratio increased steadily from 0 to 4 min, peaking at approximately 2.45 g/g, followed by a mild decline between 6 and 10 min (2.38 to 2.12 g/g). This correlates with the protonation of chitosan's amine groups and initial water penetration. After the peak, the network rearranges, strengthening hydrogen and electrostatic bonds, and expelling some free water. The addition of *Origanum vulgare* extract modulates late‐stage swelling, enhancing antioxidant capacity while reducing the leaching of lightweight components.

The rapid peak provides adequate initial hydration, while the gentle decline helps maintain mechanical integrity—behaviors consistent with responsive hydrogels for skin repair. The swelling range aligns with typical values for biopolymer‐based hydrogels, indicating improved network stability.

Sample HBSAM‐C is more favorable for rapid hydration and lasting moisture, while Sample CCO offers balanced behavior with higher structural stability and better control of free water. Together, these complementary nanocomposites can be designed for skin rejuvenation and repair systems [27].

### 3.6 Gas Chromatography‐ Mas Spectroscopy (GC–MS)

Gas Chromatography–Mass Spectrometry (GC–MS) is an analytical method that identifies compounds in a sample by combining gas chromatography and mass spectrometry. Essentially, GC–MS operates on the principle that a mixture, when heated, decomposes into distinct components. This method is one of the most essential and practical analytical systems.

GC–MS analysis was used to identify volatile compounds in two main extracts: a selenium‐rich BN extract ( *Bertholletia excelsa* ) and an oregano extract ( *Origanum vulgare* ). The results provided precise identification of chemical components, establishing a reliable basis for interpreting the biological activities and potential applications of these extracts in skin formulations.

In the BN extract analyzed (Table 2), camphor was identified as the dominant component, accounting for 49.96%, significantly higher than other groups, such as hydrocarbons (11.57%) and monoterpene hydrocarbons (19.96%). Studies indicate that nuts, including Brazil nuts, contain volatile compounds like terpenes and phenols. The high concentration of oxygenated compounds, such as Norcamphor, may interact directly with skin proteins and lipids, modulating inflammatory processes, reducing oxidative stress, and stimulating cellular regeneration. Additionally, the presence of *β*‐Pinene and Dodecane contributes to the physicochemical stability and permeability of formulations based on this extract [28] and (Table 3).

**TABLE 2:** Compounds in Brazilian nut extract from GC–MS analysis.

No.	Compound	%	RI
1	camphor	49.96	983
2	*β*‐Pinene	19.96	979
3	Dodecane	11.57	1200
**Total Identified**		**81.49**	

**TABLE 3:** Compounds in oregano essential oil.

No.	Compound	%	RI
1	Sabinene	1.06	909
2	Limonene	2.18	1030
3	Menthone	8.73	1138
4	Borneol	4.94	1154
5	α‐Terpineol	3.02	1171
6	Pulegone	14.62	1262
7	Piperitone Oxide	24.25	1271
8	Thymol	2.59	1316
9	Piperitenone	22.06	1271
10	*β*‐Caryophyllene	3.50	1467
11	Caryophyllene Oxide	4.63	1450
12	Palmitic acid	1.02	2005
Total Identified		92.6	

The high concentration of oxygenated monoterpenes, such as Piperitone oxide and Pulegone, in this sample could be valuable for skin formulations. The presence of functional oxygenated groups (alcohols, ketones, and epoxides) enables strong interactions with skin proteins and lipids, potentially conferring antioxidant, anti‐inflammatory, and tissue‐regenerative properties. This suggests that oregano essential oil, with its specific chemotype, has significant potential for developing skin products such as hydrogels and creams [29].

The BN extract, with a dominance of oxygenated compounds (49.96%) and the primary presence of Norcamphor, represents a semi‐polar profile with strong carbonyl‐protein interactions, contributing to the modulation of inflammation, reduction of oxidative stress, and stabilization of skin formulations. In contrast, oregano essential oil, with a significant dominance of oxygenated monoterpenes (80.21%), primarily featuring Piperitone oxide and Pulegone, provides a highly active combination with more potent antioxidant and anti‐inflammatory capacities [30].

In summary, the chemical profile of BN supports stability and biocompatibility in formulations. At the same time, oregano, with its high concentration of oxygenated monoterpenes, offers greater biological activity and practical reparative effects for skin rejuvenation systems.

### 3.7 Zeta Potential Analysis (ZPA) and Dynamic Light Scattering (DLS)

ZPA was conducted to evaluate the key properties of nanocomposites made from biomaterials. Two samples were analyzed, showing Zeta potentials of −18.9 mV and +17.6 mV. ZPA indicates colloidal stability; values above ±30 mV indicate excellent stability, while values between ±10 and ±30 mV suggest moderate stability. These analyses are crucial for determining colloidal stability, particle size distribution, and surface interactions (Figure 7).

In the HBSAM‐C nanocomposite, composed of hyaluronic acid (HA) and chitosan, a ZPA of −18.9 mV indicates moderate stability and effective negative charge due to carboxyl groups in HA, which prevent particle aggregation. Dynamic Light Scattering (DLS) revealed particles < 200 nm in size, with uniform dispersion, highlighting HA's role in moisture retention and physical stability [22].

In the CCO nanocomposite lacking chitosan and loaded with oregano extract, the Zeta potential was +17.6 mV. This positive charge likely comes from bioactive compounds in oregano, enhancing surface interactions with cell membranes and skin absorption. DLS confirmed particles within the nanometric range, indicating stability without additional cationic polymers [31] and (Figure 7a,b).

Both systems fall within a stable range (±10 to ±30 mV), with HBSAM‐C showing greater physicochemical stability. At the same time, CCO, due to its positive charge, exhibits greater biocompatibility and potential for skin interaction.

### 3.8 Release of Ascorbic Acid From Nanocomposites

In the first nanocomposite sample, a release test was conducted in a simulated physiological environment to investigate the mechanism and extent of ascorbic acid release. For this purpose, dry samples of the HBSAM‐C nanocomposite were placed in semi‐permeable membrane bags to prevent leakage of polysaccharide particles while allowing ascorbic acid molecules to pass freely. The main compound is gradually released into the environment.

A precise amount of 15 mg of the dry nanocomposite was placed in each membrane bag and maintained in a beaker containing 50 mL of PBS (pH 7.4) at 37°C ± 0.5°C and 100 rpm. Samples were taken at 0.5, 1, 2, 4, 8, 24, 48, and 72 h, with 3 mL of solution withdrawn each time and replaced with an equal volume of PBS to maintain sink conditions. The absorbance of the samples was measured at the wavelength λ_max_ = 265 nm using a UV–Vis spectrophotometer. The selected sample amount and medium volume were adjusted to achieve a 50% weight loading of ascorbic acid, given its high solubility in PBS, optimizing optical sensitivity while maintaining sink conditions.

The absorbance spectrum of the samples across 200–800 nm showed a distinct peak at 265 nm, indicating effective release of ascorbic acid in PBS. The absence of secondary peaks in the visible region (400–800 nm) showed that there were no spectral interferences from the phenolic compounds in Brazilian almond extract, confirming that the absorbance readings were exclusively due to vitamin C.

In the time curves, the initial phase exhibited a rapid increase in absorbance, attributed to burst release, caused by the quick detachment of surface molecules of ascorbic acid from the polysaccharide matrix. In subsequent stages, the slope of the graph decreased, indicating that Fickian diffusion controlled the release within the moist matrix.

The presence of Brazilian almond extract in the nanocomposite structure resulted in a slight reduction in the release rate and increased uniformity. The antioxidant and relatively hydrophobic properties of this extract limited water absorption, reduced swelling, and stabilized the matrix structure in the moist phase. This characteristic contributed to the stability of vitamin C and prevented its rapid oxidation.

In the CCO nanocomposite, to accurately determine release kinetics and evaluate its performance, the release profile of phenolic active compounds in a simulated physiological environment (PBS, pH 7.4, 37°C) was investigated. 15 mg of dry nanocomposite was precisely weighed and placed in a 50 mL PBS solution, which was stirred at 100 rpm.

The release monitoring was performed at specified time intervals using UV–Vis spectrophotometry at λ_max_ = 265 nm (the characteristic peak of phenolic compounds). Sequential sampling, along with the replacement of an equivalent volume of PBS, maintained sink conditions throughout the test [32] and (Figure 7C).

The absorbance spectrum of the sample over 200–800 nm indicates a stable, transparent optical structure in the nanocomposite matrix. The ultraviolet region (200–400 nm) contains a main peak at 265 nm, clearly associated with the π → π* electronic transition of the active phenolic compounds from the oregano extract. The gradual decline in absorbance at higher wavelengths suggests high optical stability and the absence of oxidation of active compounds in the moist phase. The lack of secondary peaks in the visible region (400–800 nm) confirms the absence of phenolic interference, the nanocomposite's stability, and the PBS medium's complete transparency [32].

### 3.9 Bacterial Growth Curves in MRS Broth

The growth curve of two bacterial species, *B. subtilis* and *L. plantarum* , in MRS broth was examined over 8 h. The graph shows that changes in optical density (OD) during different growth phases indicated differences in growth rates and survival between the two species. *Lag Phase* (0–2 h): The increase in OD was minimal, indicating the cells' initial adaptation to the new environment. *Exponential Phase* (2–4 h): *Bacillus* showed a higher growth rate (OD = 1.79) than ***L. plantarum*** (OD = 1.41). This difference can be attributed to *B. subtilis* higher ability to exploit environmental resources quickly. *Stationary* Phase (4–6 h): The optical density of both species remained approximately constant, indicating they had reached their final growth capacity in those environmental conditions. *Death Phase* (6–8 h): The decrease in OD for *B. subtilis* was more pronounced than that of *L. plantarum* (0.735 vs. 0.829), suggesting greater sensitivity of *B. subtilis* to environmental stresses and resource depletion. In contrast, *L. plantarum* exhibited a slower death rate, indicating higher tolerance to adverse conditions.

Studies on *L. plantarum* FNCC 0026 also reported that this species reached its highest OD in MRS broth after about 30 h, after which it entered the stationary phase. In this study, the peak of antibacterial activity coincided with the stationary phase [33]. It was also found that *L. plantarum* AC 11S exhibited stable growth and a more extended lag phase compared to the control in mMRS broth after an acid shock [3].

*B. subtilis* is a species with rapid growth but short‐term stability, while *L. plantarum* grows more slowly but exhibits higher biological stability and environmental resistance. Therefore, in skin rejuvenation formulations, *L. plantarum* is a more suitable and functional choice.

### 3.10 Gram Staining in Probiotic Bacteria

Gram staining, one of the most precise differential methods for identifying bacterial cell wall type, was used in this section to confirm the morphology, physiological health, and purity of selected probiotic strains ( *L. acidophilus* and *L. plantarum* ).

According to Figure 7d, purple bacilli, observed in both single and chain arrangements under a light microscope, confirmed the presence of a thick peptidoglycan layer and the Gram‐positive characteristics of these bacteria. These results not only demonstrate the purity of the culture and accuracy of microbial isolation but also confirm the compatibility of these strains with laboratory environments and the conditions of the nanocomposite formulation.

The high stability of the positive Gram bacteria used in this research, along with their successful staining, indicates their compatibility with laboratory environments and the successful biological performance of the nanocomposites in drug development pathways. These findings align with international scientific literature.

### 3.11 Antibacterial Activity

In examining the antibacterial potency of the synthesized nanoparticles against four clinical bacterial strains— *S. aureus* , *E. faecalis* , *P. aeruginosa* , and *E. coli—*the results indicated that the nanoparticles exerted significant antibacterial activity only against Gram‐positive bacteria.

The results in that the synthesized CCO nanocomposite exhibits antibacterial activity against *S. aureus* and *E. faecalis* , with MIC and MBC values of 62.5 μg/mL. However, it shows no antibacterial effects against the Gram‐negative bacteria *P. aeruginosa* and *E. coli* , indicating its specificity for Gram‐positive strains.

For the *S. aureus* strain, the MIC and MBC values were 62.5 μg/mL in the initial sample and 31.25 μg/mL in the secondary sample. These results demonstrate that both growth inhibition and lethal effects were observed for *S. aureus* . Still, the efficacy was higher in the secondary sample, which may be attributed to slight differences in nanoparticle synthesis or chemical properties.

In the case of *E. faecalis* , the MIC was 62.5 μg/mL in both samples, but no MBC was observed, indicating that the nanoparticles exerted only a growth‐inhibitory effect without a lethal definitive impact. This event highlights the distinction between the bacteriostatic and bactericidal activities of nanoparticles across different strains.

Notably, no specific MIC or MBC values were recorded for the 2 Gram‐negative bacteria, *E. coli* and *P. aeruginosa* , indicating that these nanoparticles had no significant effect on the growth or survival of these strains. These results are consistent with studies reporting the efficacy of nanoparticles primarily against Gram‐positive bacteria, with the unique physiological resistance and structure of Gram‐negative bacteria, including the outer lipopolysaccharide layer and efflux pumps, being the main reasons for this.

In the disk diffusion test, the inhibition zone diameter for *S. aureus* was 11 ± 0.5 mm and 15 ± 0.5 mm for the initial and secondary samples, respectively. Although lower than the positive control (gentamicin and ciprofloxacin, with inhibition zone diameters of 22–26 mm), the effect of this nanocomposite on Gram‐positive bacteria is reasonably confirmed. For *E. faecalis* , the inhibition zone diameter was significantly smaller, around 7–11 mm, whereas for Gram‐negative bacteria, very low or even zero values were obtained, reiterating the importance of cell wall structure and the inherent defense capabilities of Gram‐negative bacteria.

Figure 7e illustrates the inhibition zones produced by the synthesized nanocomposites against various bacterial strains, including *S. aureus, E. faecalis, E. coli*, and *P. aeruginosa* . The varying diameters of the inhibition zones demonstrate the nanocomposites' effectiveness in inhibiting bacterial growth.

In conclusion, the results clearly support the scientific fact that the studied nanoparticles exhibit both bacteriostatic and bactericidal effects on Gram‐positive bacteria through various mechanisms and could be considered suitable candidates for practical application in treating infections caused by this group, while their role against Gram‐negative agents is limited unless combined with structural modifications or other antimicrobial agents (Slavin et al., 2017 [34]). These findings align well with the values reported in articles such as Rai et al. (2009) [35], reinforcing their scientific validity. The methods and criteria for determining MIC and MBC also conform to accepted global standards, providing reliable and credible results for future similar assessments.

The second sample demonstrated greater bactericidal activity against *S. aureus* and a larger inhibition zone. At the same time, its effect on *E. faecalis* was limited to growth inhibition, with no activity against Gram‐negative bacteria. The enhanced response of Gram‐positive bacteria results from the greater ease with which nanoparticles penetrate the peptidoglycan structure. Thus, the second sample is evaluated as a more effective candidate for treating Gram‐positive infections, particularly *S. aureus* .

Figure 7e shows that the HBSAM‐C nanocomposite exhibits strong bactericidal activity against *S. aureus* and limited growth inhibition of *E. faecalis* , with no effect on Gram‐negative bacteria. Figure 7f indicates that the CCO nanocomposite similarly targets *S. aureus* , highlighting its potential for treating Gram‐positive infections.

### 3.12 Analysis of Nanocomposite Ointment Based on Nanohydrogel

A nanocomposite ointment based on nanohydrogel was successfully prepared using an oil‐in‐water (O/W) emulsification method. The final formulation appeared as a uniform, cream‐like, off‐white mixture with no signs of phase separation or sedimentation. The final pH was 5.5 ± 0.2, within the physiological range (Figure 7g).

Initial stability tests indicated that the formulation remained stable for at least 4 weeks at room temperature and in refrigeration, with no significant changes in consistency, color, pH, or phase separation. The inclusion of petrolatum as an oil base, along with vitamin E, enhanced occlusivity and moisture retention while also improving chemical stability and oxidation resistance.

Comparative analysis with recent studies shows that this method is competitive in terms of simplicity, energy consumption, and final efficiency. For instance, Mehmood et al. (2024) [36] reported a formulation using solid lipid nanoparticles (SLN) based on hyaluronic acid and tretinoin, which required a more complex emulsification‐diffusion technique resulting in larger particle sizes and a zeta potential of +13 mV, indicating acceptable colloidal stability but larger particle sizes compared to the nanohydrogel structure developed in this study.

Overall, this method successfully maintains process simplicity while yielding a formulation with desirable physicochemical properties, including physiological pH, uniform appearance, and acceptable initial stability. These characteristics make it highly suitable for developing therapeutic or cosmetic products based on nanohydrogel with moisturizing, anti‐inflammatory, and antioxidant properties.

## 4 Conclusion

In this study, innovative hydrogel nanocomposites were successfully developed using natural, biocompatible materials for effective skin rejuvenation. Two formulations, HBSAM‐C and CCO, were designed to combat both internal and external aging factors, using components such as hyaluronic acid, BN extract, chitosan, and *Origanum vulgare* extract. The formulations demonstrated significant moisture absorption, strong antibacterial activity against Gram‐positive bacteria, and favorable physicochemical properties.

Statistical analyses using Response Surface Methodology (RSM) confirmed the influence of formulation variables on particle size and stability. The findings indicate that the developed nanocomposites are suitable for therapeutic applications in skin care due to their biocompatibility, effective release of active compounds, and potential for enhancing skin health.

Future research is encouraged to explore integrating these nanocomposites into topical formulations, such as serums, lotions, and sunscreens, to leverage their beneficial properties for skin rejuvenation further. Overall, this work highlights the promising applications of hydrogel nanocomposites in dermatological products that aim to improve skin elasticity, hydration, and overall appearance.

## Funding

This research received no specific grant from funding agencies in the public, commercial, or not‐for‐profit sectors.

## Ethics Statement

The authors have nothing to report. Approval Number/Code: IR.IAU.TNB.REC.1403.166. Name of Approving Ethics Committee or Board: Islamic Azad University—North Tehran Branch (Research Ethics Committee). Informed Consent Statement: No animal studies were conducted in this research.

## Consent

All authors have given their consent for the publication of this manuscript and its associated materials.

## Conflicts of Interest

The authors declare no conflicts of interest.

## Data Availability

Research data are not shared.
